# A New Approach to Estimating the Parameters of Structural Formations in HDPE Reactor Powder

**DOI:** 10.3390/polym15183742

**Published:** 2023-09-13

**Authors:** Artem Borisov, Yuri Boiko, Svetlana Gureva, Ksenia Danilova, Victor Egorov, Elena Ivan’kova, Vyacheslav Marikhin, Liubov Myasnikova, Ludmila Novokshonova, Elena Radovanova, Elena Starchak, Tatiana Ushakova, Maria Yagovkina

**Affiliations:** 1 Laboratory of Physics of Strength, Ioffe Institute, Polytechnicheskaya St. 26, St. Petersburg 194021, Russia; borisov.ak@mail.ioffe.ru (A.B.); swet.gurjewa@gmail.com (S.G.); ksdanilova@bk.ru (K.D.); victor_egorov1@inbox.ru (V.E.); v.marikhin@mail.ioffe.ru (V.M.); radeli@mail.ioffe.ru (E.R.); ymasha@mail.ioffe.ru (M.Y.); 2 Institute of Macromolecular Compounds, Bol’shoy pr. 31, St. Petersburg 199004, Russia; ivelen@mail.ru; 3 Semenov Institute of Chemical Physics, Kosygina St. 4, Building 1, Moscow 119991, Russia; lnov@chph.ras.ru (L.N.); star2004i341@rambler.ru (E.S.); tmush2017@yandex.ru (T.U.)

**Keywords:** high-density polyethylene (HDPE), reactor powder, small-angle X-ray scattering (SAXS), wide-angle X-ray scattering (WAXS), differential scanning calorimetry (DSC), scanning electron microscopy (SEM), long period, crystallinity, lamella, shish kebab

## Abstract

The morphology of virgin reactor powder (RP) of high-density polyethylene (HDPE) with M_W_ = 160,000 g/mol was investigated using DSC, SEM, SAXS, and WAXS methods. The morphological SEM analysis showed that the main morphological units of RP are macro- and micro-shish-kebab structures with significantly different geometric dimensions, as well as individual lamellae of folded chain crystals. A quantitative analysis of an asymmetric SAXS reflection made it possible to reveal the presence of several periodic morphoses in the RP with long periods ranging from 20 nm to 60 nm, and to correlate them with the observed powder morphology. According to the DSC crystallinity data, the thickness of the lamellae in each long period was estimated. Their surface energy was calculated in the framework of the Gibbs—Thompson theory. The presence of regular and irregular folds on the surface of different shish-kebab lamellae was discussed. The percentage of identified morphoses in the RP was calculated. It has been suggested that the specific structure of HDPE RP is due to the peculiarity of polymer crystallization during suspension synthesis in a quasi-stationary regime, in which local overheating and inhomogeneous distribution of shear stresses in a chemical reactor are possible.

## 1. Introduction

Ultra-high molecular weight polyethylene (UHMWPE) continues to be one of the most popular and widely used polymer materials due to its high chemical resistance to aggressive media, high wear resistance, low friction coefficient, high impact strength, fiber formation ability, and the possibility of obtaining high performance fibers, on the basis of which products for various purposes are created: from bulletproof vests and armor to boat hulls, towing ropes, medical implants, suture material, etc. As a result of the peculiarities of the supramolecular structure of UHMWPE, characterized by the presence of a large number of entangled tie molecules in disordered intercrystalline and interlamellar regions of the material, UHMWPE does not go into a viscous-liquid state even at temperatures exceeding the melting point of crystallites [1]. This polyolefin has a very high melt viscosity, which makes it impossible to process it using traditional methods such as ram extrusion and injection molding. Therefore, for the processing of UHMWPE, for example, into high-strength, high-modulus fibers, a gel technology method was developed, which consists of spinning a low-concentration polymer solution in non-polar solvents (decalin, mineral oil a.o.) followed by orientation drawing [2]. However, this method is expensive due to the necessary recuperation of large volumes of solvent and it is also ecologically non-friendly. In recent years, a solution-free (“dry”) method for processing UHMWPE directly from a reactor powder (RP), similar to the process of powder metallurgy, has been actively developed. This method consists of sintering powder particles into a mechanically coherent film at a temperature below the melting point of the polymer, which is then subjected to orientation hardening [3,4,5,6,7,8,9]. However, if the nascent structure of the reactor powder used in gel technology is not of great importance because it is practically totally destroyed during solving, and a new structure is formed during gel crystallization, the requirements of the reactor powder morphology increase tremendously when solution-free processing is used.

The development of solid-state processing resulted in intensive studies of reactor powder morphologies, as well as the exploration of new catalytic systems designed to obtain a synthesis product with minimal entanglements in the disordered regions. These entanglements control the compactability and drawability of the powder [10,11,12,13,14].

The sintering of reactor powders with subsequent high-temperature orientation drawing remains a much more acceptable method for processing UHMWPE high performance fibers. However, development high performance fibers is limited by dependence on the proper reactor powder.

At the same time, the searching for other routes to obtain cheaper and more reliable UHMWPE processing continues.

On one hand, attempts have been made to process UHMWPE in its molten state using modified methods of ram extrusion and compression molding, involving a significant increase in temperature (up to 200 °C) and an increase in the residence time of the polymer at elevated temperatures (up to 2 h) [15]. However, the presence of the polymer for a long time at a high temperature inevitably leads to its destruction and to a deterioration of its properties.

The other method for improving the technological properties of UHMWPE is to create mixtures with a wide bimodal molecular weight distribution by introducing polyolefins with a high melt fluidity into the matrix [16,17,18,19,20,21,22,23,24,25,26]. The use of low molecular weight HDPEs (LMWPEs) as components in mixtures based on UHMWPE is relevant and promising due to the similarity of the structures of UHMWPE and LMWPE molecular chains. Interest in polymer blends has been steadily increasing over the past few years [16,17,18,19,20,21,22,23,24,25,26]. Mixing two or more matrices can create new polymers with improved properties, different from their constituents, and can overcome the traditional problems that arise when synthesizing new polymer species.

Many authors have reported on the compatibility of UHMWPE/LMWPE components. However, the large discrepancy in the viscosity of the melts of the components is the reason the traditional method of mechanical mixing in the melt leads to poorly dispersed mixtures with a UHMWPE fraction content of ≥10 wt.%. For instance, studies have been carried out on the HDPE/UHMWPE blends obtained by melt blending, with UHMWPE concentrations ranging from 10 to 30% by weight, using an intermeshing co-rotating twin screw extruder [20]. SEM analyses of the morphology of such blends show that UHMWPE forms a separate phase in the HDPE matrix with a good interface. In addition, HDPE/UHMWPE blends with a UHMWPE content of up to 20% by weight have been prepared using a single screw extruder or an internal mixer as a function of the mixing time [23]. The evaluation of the melt viscosity of these blends indicated a slight dissolution of UHMWPE and its presence as a filler.

Undoubtedly, most methods of mixing polymers have their limitations and require special conditions in order to avoid phase separation of blends.

The most effective and relevant for the creation of polymer compositions based on UHMWPE is the use of “reactor” methods that allow polyolefin fractions to be introduced into its matrix directly during the polymerization of ethylene on organometallic catalysts. These methods include two-stage polymerization processes using the same catalytic system at each stage [27,28,29]. Compositions based on UHMWPE and linear LMWPE with different contents of low-molecular fractions were obtained through two-stage polymerization of ethylene over a homogeneous zirconocen catalyst when the synthesis conditions changed during the stages of the process. With an increase or decrease in polymerization temperature, the ratio of the growth reaction rate of the polymer chain and the sum of the growth restriction reaction rates of the polymer chain changed; as a result, there was a decrease or increase in the molecular weight of HDPE, respectively [30,31,32].

The synthesis of the UHMWPE fraction with M_w_ = 1000 kg/mol and MWD = 3 was carried out at a polymerization temperature of 30 °C, and the LMWPE fraction with M_w_ = 160 kg/mol and MWD = 2.8 at 70 °C. The compositions UHMWPE/LMWPE had high tensile properties exceeding the properties of unmodified UHMWPE. At the same time, there was an increase in the fluidity of the material; a number of UHMWPE reactor compositions with LMWPE flowed at loads of 10.5 and 5 kg with MIs of 0.52 and 0.03 g/10 min, respectively, which ensured the processing of materials by traditional methods [29,33].

When searching for proper compositions of UHMWPE/LMWPE with desirable properties, detailed information on the fine structure of the nascent particles both of UHMWPE and LMWPE are needed. The morphology of UHMWPE reactor powders synthesized on various catalytic systems and synthesized, in particular, on new metallocene catalysts, has been widely studied in recent decades in connection with the active development of solid-phase processing of UHMWPE. The nascent structure of LMWPE reactor powders was studied even earlier. A large amount of significant data have been obtained. Most research works are devoted to the mathematical modeling of molecular growth and crystallization during synthesis, catalyst fragmentation, the effect of synthesis temperature on crystallization, etc. [34,35,36,37]. In the process of synthesis, nonequilibrium crystallization occurs, which differs from crystallization from a quiescent melt or solution. In the latter case, the thickness of the lamellae always increases with increasing T, and in the process of synthesis, an inverse relationship is observed—with an increase in the synthesis temperature, the thickness of the lamellae decreases, which confirms the Hoffmann-Lauritzen kinetic theory of crystallization. Usually, LMWPEs are synthesized on supported Ziegler-Natta catalysts or by gas-phase polymerization. We are not aware of studies on the morphology of HDPE reactor powders synthesized on metallocene catalysts. At the same time, for two-stage synthesis on a metallocene catalyst, HDPE is initially synthesized, and only then, after lowering the reactor temperature, does UHMWPE begin to grow on it. Knowledge about the structure of the polymer formed in the first stage is very important.

So, the aim of this work is to study the complicated hierarchical structure of nascent HDPE at all levels of molecular organization.

## 2. Materials and Methods

### 2.1. Objects of Study

For this study, HDPE reactor powder with an average molecular weight (Mw) of 160,000 g/mol, MWD = 2.8 (HDPE), synthesized at the Federal Research Center for Chemical Physics, named after N.N. Semenov RAS, was chosen. It was used as a low-molecular fraction in reactor compositions based on UHMWPE with M_w_ = 1000,000 g/mol and MWD = 3. The absorption bands at 1380 cm^−1^ in the IR spectra of these polymers were absent. This means that the amount of CH_3_ branching was small, comprising <0.5 CH_3_/1000C, and that both polymers were linear.

The synthesis of UHMWPE/HDPE reactor compositions was carried out using a two-stage sequential polymerization of ethylene using a metallocene catalyst rac-(Me)_2_Si(Ind)_2_ZrCl_2_/methylalumoxane, with varying polymerization temperatures at each individual stages. The temperature of the glass reactor was maintained by thermostat water flowing between the double walls of the reactor. The volume of the reactor was 0.4 L. Toluene was used as the solvent; the concentration of ethylene was 9.2 × 10^−2^ mol/L.

During the first stage of the process, at a polymerization temperature of 70 °C, the HDPE fraction of compositions was synthesized and sampled for further studies. The UHMWPE fraction was obtained during the second stage of the process at a temperature of 30 °C in the presence of the HDPE suspension containing a catalytic complex. The structure of HDPE can influence the composition of UHMWPE/HDPE.

### 2.2. Methods

#### 2.2.1. Scanning Electron Microscopy

The morphology of the powder particles was studied using a Zeiss Supra 55VP (Zeiss, Jena, Germany) scanning electron microscope. To avoid charge accumulation when scanning samples with an electron probe, the particles were placed on a conductive substrate and a thin layer of platinum was deposited onto them using an Q150T ES (Quorum Instruments, Laughton, UK).

#### 2.2.2. Thermal Analysis

Thermograms were recorded on a DSC-500 differential scanning calorimeter (Spetspribor, Samara). The temperature scale was calibrated using the melting points of ice (273.1 K) and indium (429.7 K), and the heat flux was calibrated using the heat capacity of sapphire. The scanning speed (V) varied from 1.0 to 10 K/min. The true melting temperature (T^t^_m_), as well as the true temperatures of the beginning (T^t^_1_) and end (T^t^_2_) of melting, were obtained by extrapolating the dependences of T_m_(V^1/2^), T_1_(V^1/2^), and T_2_(V^1/2^) to a zero-heating rate (V = 0), respectively [38]. According to this extrapolation, the methodological error ΔT was estimated, which, in this case, was ΔT = 1.6 K; this was taken into account in the calculations. The melting enthalpy (ΔH_m_) was calculated from the area of the melting peak, and the degree of crystallinity χ was calculated from the relation χ = ΔH_m_/ΔH^0^_m_, where ΔH^0^_m_ is the melting enthalpy of an ideal PE crystal (4.1 kJ/mol = 294 J/g) [39].

#### 2.2.3. X-ray Diffraction Analysis at Small Angles (SAXS)

Small-angle X-ray scattering (SAXS) curves from the powder were recorded for reflection in the θ–2θ scanning mode (angle range 0 ≤ 2θ ≤ 2) on a Bruker D8 Discover diffractometer equipped with a rotating anode as an X-ray source. We used monochromatized CuK_α1_ radiation with a wavelength of λ = 1.5406 Å. The reactor powder was placed in a standard cuvette and a drop of Vaseline oil, with an electron density close to the electron density of polyethylene, was added in order to create a flat surface of the sample so as to reduce diffuse X-ray scattering on a rough surface and to suppress scattering on the pores. The resulting small-angle scattering curve was decomposed into elementary peaks using the Fityk 1.3.1 program, and for each peak, the value of the long period L_SAXS_ was calculated. Scientific articles concerned with the study of the supermolecular structure of reactor PE powders usually describe the absence of a regular long period structure. In fact, this is due to the masking effect of X-ray scattering in the air in the pores of reactor particles with various loosely packed morphological units.

#### 2.2.4. X-ray Diffraction Analysis at Large Angles (WAXS)

X-ray diffractometry in the region of large angles from the studied HDPE reactor powder was carried out on a Bruker 2DPhaser powder diffractometer using a PSD detector and a nickel ß-filter (CuK_a1_ radiation, λ = 1.54 Å) at a voltage of 30 kV and current of 10 mA. Registration was carried out in steps of 0.036 degrees in the angular range of 60–84 degrees on the 2θ scale. The curve was recorded in the accumulation mode for 24 h. The average sizes of crystallites in the direction perpendicular to the plane (D_002_) were calculated from the linear half-widths of the 002 reflection (Scherrer method).

## 3. Results and Dicussion

### 3.1. Scanning Electron Microscopy

**Figure 1:**
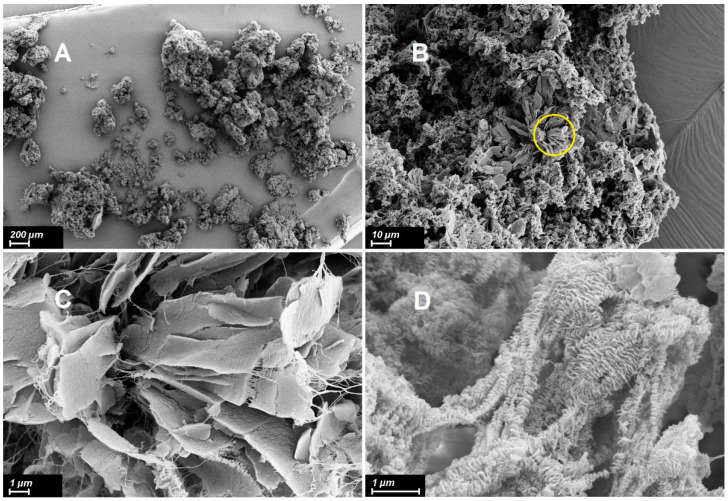
Scanning electron micrographs of HDPE (**A**–**D**) at various magnifications, shown in the images with scale bars. The image of (**C**) is a close-up of the area circled in image (**B**).

**Figure 2:**
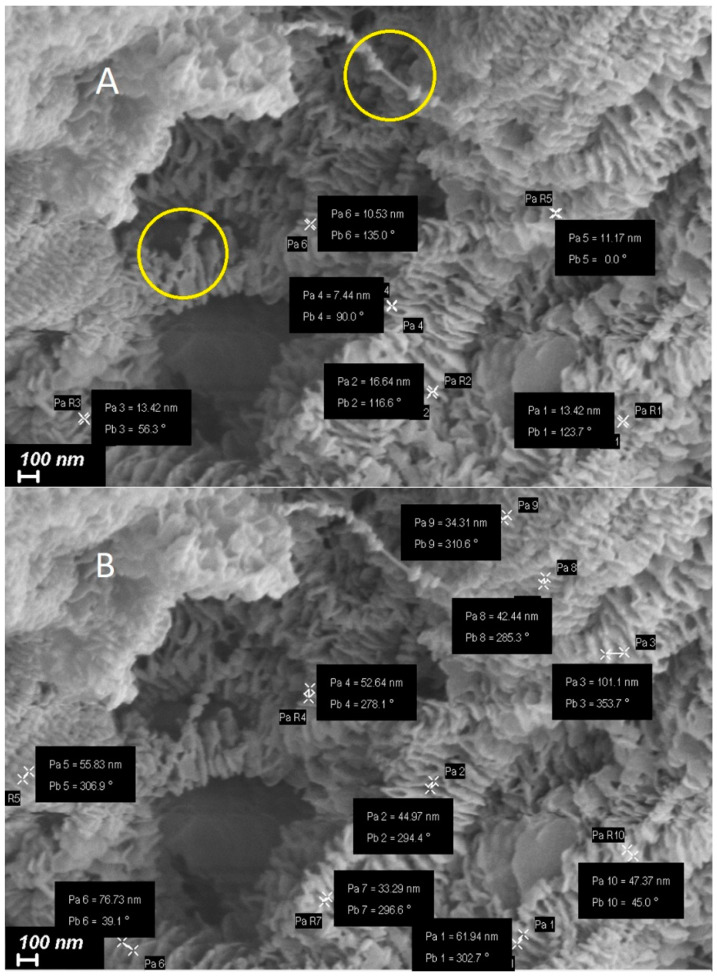
Enlarged area where shish-kebab morphological units are observed. The results of the measurements of the thickness of individual kebabs (**A**) and measurements of the periodicity in the location of kebabs on the central shishas (**B**) are shown. Central shishas are clearly visible in yellow circles.

Figure 1 and Figure 2 show the electron micrographs of the investigated reactor HDPE powder, taken at different magnifications.

It is quite clear from the micrographs that in the particles of reactor powder, various morphological structural units coexist that are clearly visible at high magnifications. It is possible to distinguish a number of morphoses that are frequently encountered:

- Extended lamellar plates (Figure 1C is the enlarged area marked with a circle in Figure 1B). As it can be seen in the selected area, the lamellae are deformed with the formation of fibrils, similar to classical single-crystal mats with simultaneous unfolding molecular folds at the transition boundary from the lamella to fibrils. It can be assumed that these are formed by folded crystallites with regular folds.
- The fibrils themselves are formed during the deformation of the lamellae under the action of tensile forces arising from the pressure of the polymer mass growing during the synthesis. The forces leading to the deformation of the lamellae do not arise from the very beginning of the synthesis, but after the accumulation of a certain mass of polymer at a certain stage of synthesis.
- Classic shish-kebab formations (Figure 1D), which, as is well-known, comprise a central shish structure with lamellar kebabs formed by folded crystals that crystallize around it [40].
- It is important to note that the size of kebabs and the distance between them vary markedly. It is possible to distinguish at least three types of structures with transverse dimensions of about 200 nm, 100 nm, and 50 nm (Figure 2).
- Central shishas are clearly visible in Figure 2A in yellow circles.

**Figure 3:**
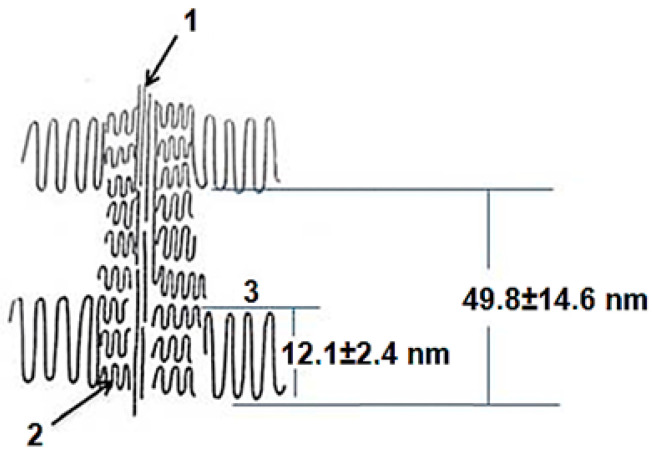
Schematic representation of the central thread (1) with micro (2) and macro (3) shish-kebab structures.

In addition, the central shish can have a complex structure and consists of a central thread formed by partially extended molecules (1) and micro-kebabs (2) growing on them (Figure 3).

The latter were not visualized on scanning micrographs. It should be noted that the micro-shish-kebab structure located in the central shish was only resolved on unstained samples of PE samples crystallized in the stirred solution using high-resolution transmission electron microscopy [40]. Thus, it is not surprising that micro-shish kebabs are not seen in SEM micrographs of the virgin particles with surfaces covered by a thin layer of platinum. It is worthy to note that the HDPE reactor powder we studied also crystallized in a stirred solution during slurry synthesis. Nevertheless, we can presumably speak of the possibility of their existence.

It is rather difficult to estimate the true percentage of the morphological units in the reactor powder using electron micrograph images. First, more statistics are needed. Second, operators often pay more attention to the most expressive structural areas and can skip areas with a less pronounced structure. Even if the operator is attentive to all possible structures, it is impossible to obtain reliable statistical results from SEM images, not to mention the fact that the ratio of different structures in the volume and on the surface of the polymer may differ. In addition, when determining the thickness of the lamellae, even in images where the lamellae are oriented sideways to the observer, there is a large uncertainty in the thickness of the platinum layer spattered to avoid charging the sample.

Nevertheless, we gathered these measurements in order to compare them with small-angle X-ray data. The average thickness of the lamellar macro-kebabs was 12.1 ± 2.4 nm, with their periodic arrangement along the central shish equal to 49.8 ± 14.6 nm. There was no polymer material between the macro-kebabs.

At the same time, scanning electron microscopy does not provide information on the distribution of crystalline and amorphous regions in morphological units. To quantify crystalline regions, disordered regions, and their location, it is necessary to involve research methods such as WAXS, SAXS, and differential scanning calorimetry (DSC).

**Figure 4:**
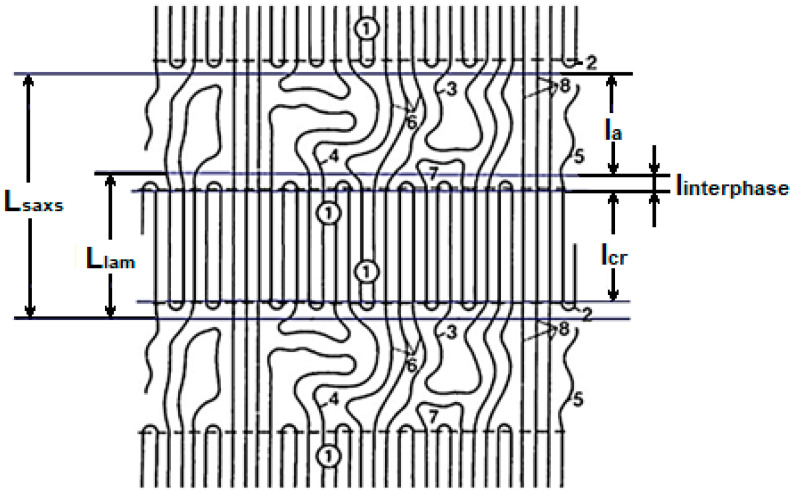
Model of a long period structure. See the designations in the text below.

Often in the literature, a long period, determined using SAXS, is denoted by the letter L. This is also used to describe the thickness of the lamella (L), which sometimes leads to some confusion in understanding the data published. To clarify the experimental data obtained, below, we present our previously proposed model for the lamellar structure [41]. The model consists of the lamellae themselves and the disordered regions separating them. The disordered regions are formed by the ends of the molecules, as well as by regular and irregular folds, along with tie molecules of different degrees of conformation (Figure 4).

L_SAXS_ is the so-called long period (l_cr_ + l_a_ + 2l_interphase_), determined by SAXS; l_cr_ (1) is the crystalline core size of the three-dimensional coherent scattering region in the direction of the chain (D_002_) determined by WAXS; l_interphase_ (2) is the transition region between the crystalline core and true amorphous region (l_a_) consisting of a set of different conformers (irregular folds (3), strongly curved molecular molecules (4), ends of molecules (5), weakly curved molecular molecules (6), molecular folds connecting 2–3 molecules at the exit of the crystallite, (7) and taut tie molecules (8); and L_lam_ is the thickness of the lamella (crystal core + two thicknesses of the more or less regular folded surface that melt simultaneously with the crystalline core). Obviously, L_SAXS_ > L_lam_ > l_cr_.

To estimate the parameters of the lamellar structure of the investigated HDPE reactor powder, the above-listed experimental methods were used.

### 3.2. Small-Angle X-ray Scattering (SAXS)

**Figure 5:**
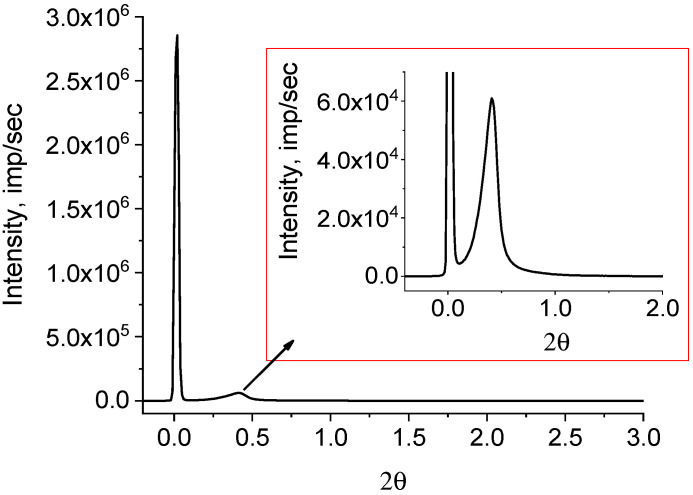
Small angle scattering from the HDPE reactor powder. The inset shows a part of the curve with a modified intensity scale (primary beam cut in height), which makes it possible to clearly observe the asymmetric diffraction maximum.

The SAXS image from the powder is presented in Figure 5.

**Figure 6:**
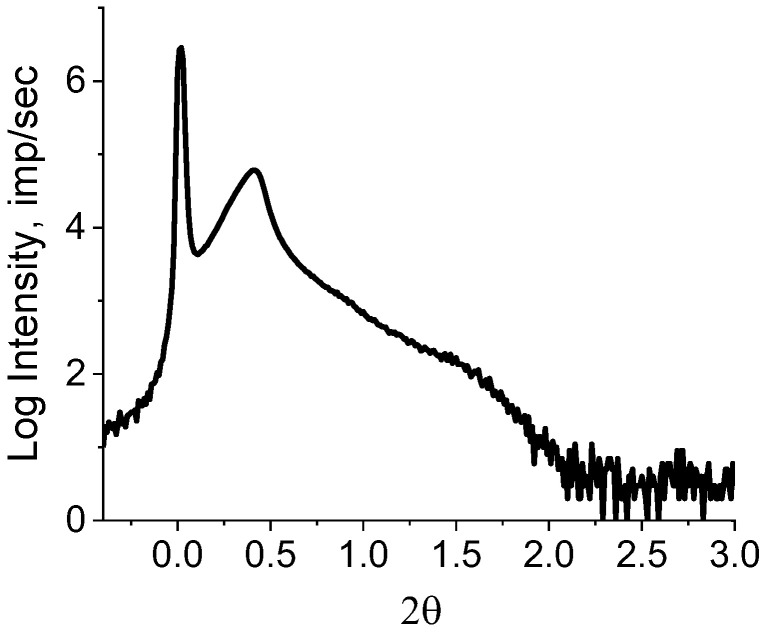
lg I(2θ) for the HDPE reactor powder.

Despite the fact that many authors, as a rule, have not detected a diffraction peak on SAXS of RP, it still exists. It is merely masked by air scattering on porous powders. We succeeded in detecting the diffraction peak by adding a drop of mineral oil to a powder sample with an electron density close to that of PE. We believe that the mineral oil filled the voids in the polymer reactor spongy-like particles through capillary action and expelled the air. If some air still remained in the powder, then scattering from it would contribute to the background intensity. As X-ray diffraction patterns were recorded in the reflection mode, the addition of mineral oil also played an important role in the smoothing sample surface. The peak is clearly shown in the inset in a right upper corner of Figure 5. The asymmetric profile of the observed peak implies the overlapping of lamellar stacks with different periodicities. It is possible to decompose the observed diffraction peak into elementary peaks with a known degree of reliability only in the range of angles of 0.1 ≤ 2θ ≤ 1.0. The intensity in the range of angles 1 ≤ 2θ ≤ 2 is small and almost does not change. When taking the logarithm of the initial curve (lg I(2θ)), a rather high intensity is observed in this region, which assumes the presence of periodic structures with other (smaller) values of long periods (Figure 6).

**Figure 7:**
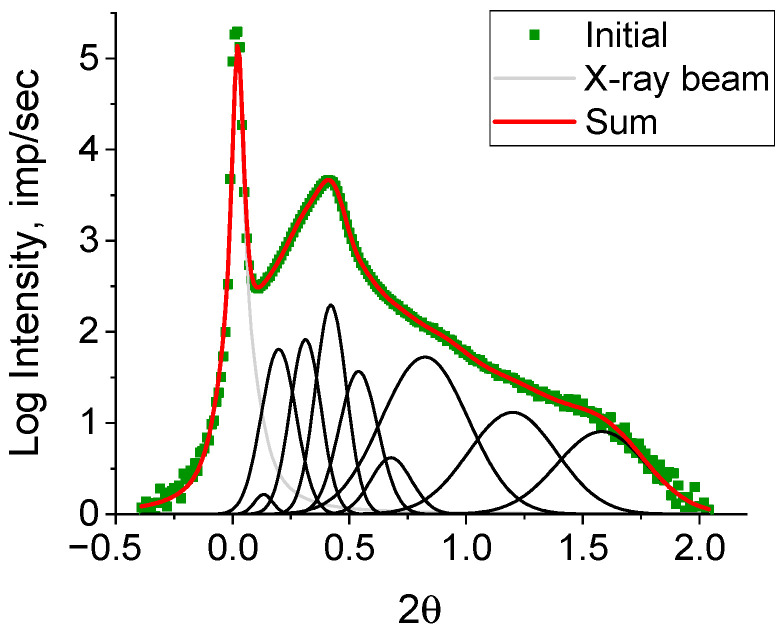
Decomposition of the dependency lg I(2θ) for the HDPE reactor powder into elementary peaks using Fityk 1.3.1 software.

The periodic structures can be identified by decomposing the resulting curve (Figure 6) using Fityk 1.3.1 software (Figure 7).

**Figure 8:**
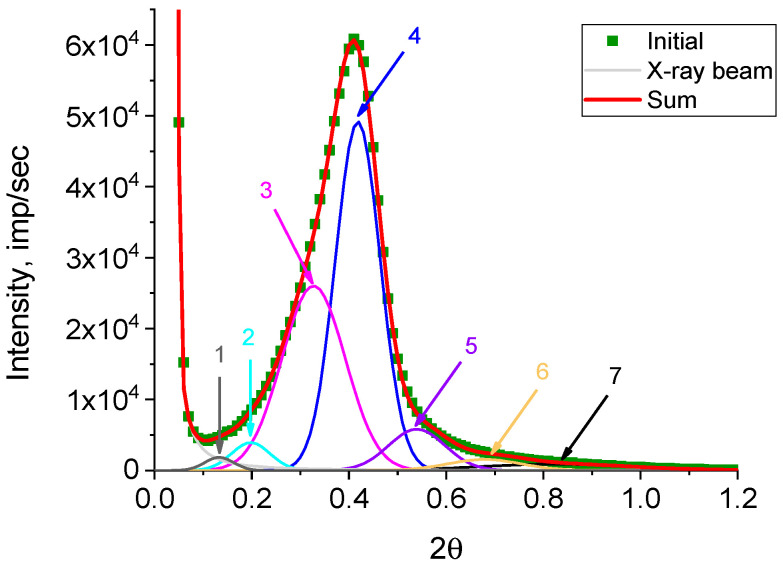
Decomposition of SAXS intensity I(2θ) of the investigated RP into separate diffraction peaks. The angular position corresponded to the angular positions of the peaks in the lg I(2θ) decomposition.

The number of substantiated peaks in the decomposition was chosen in accordance with the number of different morphoses in the powder under study. It should be emphasized that an analysis of each peak after decomposition (Figure 7) was not carried out, as the decomposition of the curve on a logarithmic scale distorted the real weight contributions of the elementary peaks to the diffraction peak. The decomposition was obtained to accurately determine the angular positions of the elementary peaks and, accordingly, the most accurate decomposition of the original curve for further analysis. To estimate the real weight contribution, decomposition of the original curve (Figure 8) was carried out with fixed angular positions of the peaks obtained by decomposing lg I(2θ) (Figure 7). As a profile function, the Pearson function was used.

**Figure 9:**
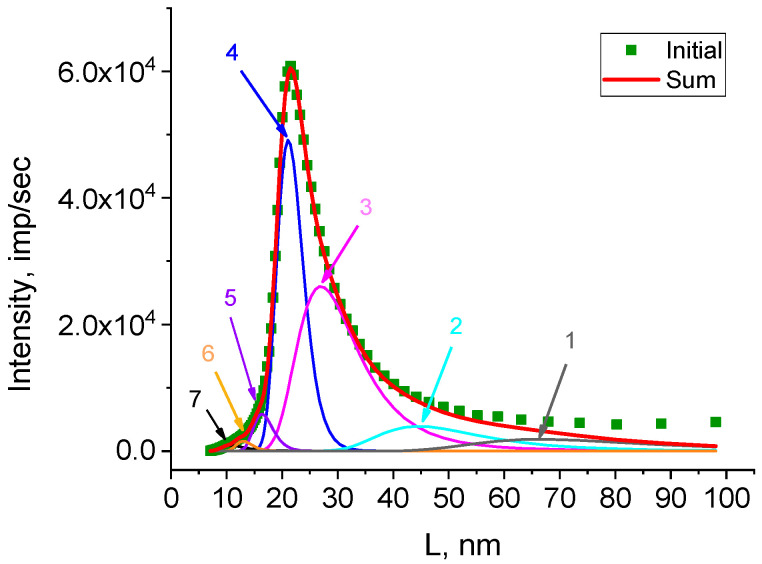
The long period (L_SAXS_) size distribution calculated from the data in Figure 8.

As is well-known, a small-angle diffraction peak occurs when a stack of alternating regions with different electron densities (regions of order and disorder) is present in the object under study, the so-called long period (L_SAXS_), as mentioned above. In our case, the regions of order were the lamellae (L_lam_) and disordered areas between them (l_a_). Moreover, the intensity of low-angle peaks depends on the squared difference in the densities (ρ) of these areas (Δρ^2^) and the degree of stack regularity (ΔL_SAXS_/L_SAXS_). The diffraction peak distribution obtained (Figure 8) was recalculated to the long period size distribution using the Bragg equation of 2Lsinθ = nλ (Figure 9).

The values of long periods L_SAXS_, calculated using the Bragg equation, and their weight contribution to the overall small-angle scattering curve in % are given in Table 1.

The comparison of the contributions of different long periods L_SAXS_ calculated from the dependence I(L_SAXS_) using SEM data allowed us to presumably attribute them to certain morphoses. For example, the large long L_SAXS_ periods (peaks 1 and 2) were closest in size to macro-shish-kebabs, clearly visible on the SEM of the HDPE microphotographs in Figure 2B (49.8 ± 14.6 nm). However, there were not many of them (7.2 and 11.0%), although the SEM images provide the impression that macro-shish kebabs were the main structural elements in reactor powders. In addition, the intensity of these peaks was low compared with the intensity of the peaks related to L_SAXS_ with a value of 21.0–27.0 nm (Figure 9). In contrast, such structures with a huge difference in density of the kebab and inter-kebab space (“voids” in SEM images) would result in the maximum scattering intensity.

Apparently, there are two reasons for the moderate contribution of these large L_SAXS_ to the total scattering curve. Firstly, the operator’s preferred choice is the shooting area with predominant localization of macro-shish-kebabs, while even in another place on the surface there may be much less of them. Secondly, large scatter in the thickness of kebabs and a scatter in the periodicity of their location along the central shish (which is clearly seen in SEM images) can significantly reduce the X-ray scattering intensity, which will lead to a decrease in the peak area and, accordingly, a decrease in the estimation of the contribution of these peaks to the overall scattering curve.

Comparing the sizes and percentages of the other elementary peaks given in Table 1 with the electron microscope images, it can be seen that the structures with a periodicity of 21.0–27.0 nm and the highest percentage (32.9 and 44.7%) were not localized along the central shish, but were inside it. These represent micro-kebabs (Figure 3), which have been mentioned above. The SEM images of the RP (Figure 2A) do not show the micro-kebabs due to the deposited metal layer; however, they still exist, apparently.

It is rather challenging to discuss the localization of structures with a periodicity of 10.7–16.3 nm, which have a very small contribution (0.6% and 2.9%). These can either be micro-kebabs with a large scatter of periodicity and/or extended lamellae, which are occasionally found on SEM images of the RP (Figure 1C).

Knowing the degree of crystallinity (χ), calculated using the area of the melting peak on the temperature-dependent heat capacity obtained by DSC, it is possible to calculate the sizes of the ordered regions. However, this method does not provide information about the regions of three-dimensional coherent scattering, which are determined using wide-angle X-ray patterns (l_cr_). Instead, DSC method considers regions that contribute to the enthalpy of melting (in our case, L_lam_ is the lamella thickness; L_lam_ = L_SAXS_ × χ) and proper disordered regions (l_a_ = L_SAXS_ − L_lam_).

The true dimensions of the crystalline core of the lamellae l_cr_ in the direction of the chain were calculated using WAXS data for the linear halfwidth of the D_002_ reflex and were 6.2 nm. Half the difference between the lamella thickness and the size of the crystalline core ((L_lam_ − l_cr_)/2) provides the value for the transition zones on both sides of the lamella, which represents the thickness of the surface layer of the lamellae (l_interphase_).

The average degree of crystallinity χ of the studied HDPE was calculated using the dependence of the heat capacity on temperature for a RP sample (m = 2.0 ± 0.1 mg) at a scanning rate of 2 K/min, was χ = 54%. However, taking into account the heterogeneity of the supermolecular structure of the studied RP, it can be assumed that the degree of crystallinity was unequal, especially for areas with a pronounced macro-shish-kebab structure. The schema in Figure 3 shows areas that are visible and invisible in microscopic images. Only macro-kebabs alternating along the thick shish can be seen. There was no polymer material between them. Obviously, the crystalline part here was much less than 54%. The “degree of crystallinity” of macro-kebabs can be estimated as follows: χ(1) = 12.1/49.8 = 24.3% (see Figure 3). At the same time, macro-kebabs accounted for 18.2% of the total set of various structures and, accordingly, somewhat underestimated the total crystallinity of the remaining structures. Thus, it was possible to estimate the crystallinity of macro-shish-kebab structures and the degree of crystallinity for all other periodic structures according to the equation below:

0.818 × χ(2) + 0.182 × 24.3 (%) = 54.0 (%),(1)

χ(2) is the crystallinity of the other structures. It was found to be 60.6%.

Thus, for macro-shish-kebab structure L_lam_ = 0.243 × L_SAXS_ and for other periodic structures L_lam_ = 0.606 × L_SAXS_.

Knowing the values of the degree of crystallinity and the melting temperature, it was possible to calculate the values of the surface energy for each lamella found from the SAXS data analysis. The true melting temperature of the investigated HDPE was determined from the heat capacity peak, taking into account the above-mentioned methodological error ΔT = 1.2 K, which amounted to T_m_ = 400.1 K. The surface energy σ_e_ was calculated using the generalized Gibbs−Thomson equation [42], based on the balance of surface and volume energies

T_m_(L) = T_0_ [1 − 2(σ/a + σ/b + σ_e_/L)/ΔH_0_], (2)

where a and b are the dimensions of the crystallite (the region of coherent scattering) in the plane of the section perpendicular to the longitudinal axis coinciding with the direction of the macromolecule, L is the longitudinal size of the crystallite, σ is the surface energy of the side surfaces of the crystallite, σ_e_ is the surface energy of the fold surface, and ΔH_0_ is the heat of fusion.

In the supermolecular lamellar structure of the polymer, parameters a and b >> L; therefore, in Equation (2), terms σ/a and σ/b can be neglected and it can be re-written in a simpler form:

T_m_(L) = T_0_ [1 − 2 σ_e_ /(ΔH_0_ L)] (3)

(4) $$\mathrm{or}\;{\;\sigma }_{e\;}=\frac{\Delta H_{0}L_{\mathrm{lam}}}{2}\times \left(1-\frac{T_{m}}{T_{0}}\right)\;$$

The following data were used in further calculations: T_0_ = 415.5 K; ΔH_0_ = 279 J/g [39]. As the long period L_SAXS_ was determined by SAXS, the longitudinal size of the crystalline part, more precisely the thickness of the lamella L_lam_ in Equation (4), was L_lam_ = χ × L_SAXS_. Table 2 shows the parameters of the structures identified in the analysis of SAXS data and their surface energies calculated using Equation (4).

Using the data in Table 2, the surface energy value (85.3 × 10^−3^ J/m^2^), which was closest to the energy of a regular fold (90 × 10^−3^ J/m^2^) [39], was observed in the thickest lamellae, with a thickness of 16.5 nm, which we assumed were localized in micro-kebabs and provided the largest contribution to the small-angle scattering curve (44.7%). At the same time, the surface energy of the lamellae with almost the same thickness (L_lam_ = 15.2 nm), which formed kebabs in macro-shish-kebab structures, also had a fairly high surface energy (78.6 × 10^−3^ J/m^2^), which allowed us to assume a similar structure for the surface of the morphoses under consideration.

However, it is surprising that in these highly perfected micro- and macro-shish formations with predominantly regular folds, the largest transition phase was observed (4.50 nm and 5.15 nm), in the context of the Gibbs−Thompson theory. The only explanation for this phenomenon can be the occurrence of strong distortions in the crystalline cores of the lamellae in the region of regular folds, which distorted the crystallographic lattice in the near-surface layers of the lamellae.

In crystalline cores of lamellae with lower values of surface energy (30–60 × 10^−3^ J/m^2^) and, accordingly, with a large number of irregular folds, such distortions were practically absent and the size of the transition zone near the lamella surface was smaller (1.85–3.35 nm).

It should be noted that the investigated HDPE reactor powder was obtained by suspension polymerization in toluene at a temperature of 70 °C and intensive stirring of the reaction solution. The stirring speed was 3000 rpm. Consequently, the crystallization of growing molecules occurred in a shear field, which differed significantly from crystallization from a melt or solution and led to shish-kebab formations. Polymerization and crystallization are exothermic processes. Despite the intensive stirring of the reactor solution, there could be local temperature regions that differ from the average temperature of the reactor.

In addition, the emergence of a crystallization nucleus from segments of extended molecules (in shishas) led to an instantaneous stress drop in the nearest environment [43,44], which allowed folded crystals (kebabs) to crystallize in a quasi-stationary field. When studying the structure of reactor powders of high and ultra-high molecular weight PE synthesized on Ziegler-Natta catalysts in slurry process, the authors [45] found an inverse dependence of lamella thickness on temperature, which decreased rather than increased with increased temperature, which confirmed Lauritzen-Hofmann’s kinetic theory of crystallization. Thus, one can suggest that the smallest micro-shish kebabs incorporated in the central shish were first formed in the region of the local enhanced temperature. Thus, the crystallization of the resulting polymer occurred in shear fields with an inhomogeneous distribution of both shear stresses and temperature over the reactor volume. This, apparently, is the reason for such heterogeneity in the size of the lamellae and for the difference in the structure of their surfaces.

## 4. Conclusions

We employed a complex of SEM, SAXS, WAXS, and DSC methods to perform a detailed study of the complex hierarchical structure of HDPE reactor powder with a molecular weight of M_W_ = 160,000 g/mol, which was synthesized at the first stage of a two-stage polymerization process of ethylene utilizing a homogeneous zirconocen catalyst. Based on the analysis of the all of the data obtained, it was concluded that the main morphological units in the powder were macro- and micro-shish-kebab formations. Although the micro-shish-kebab formations were not visualized under a scanning electron microscope, their existence was indicated by small-angle X-ray scattering data. Micro-shish-kebab formations are part of the central shish, and macro-kebabs are, similar to a culinary shish-kebab, strung on this complex central shish and separated by an empty space. Kebabs (lamellae) have different thicknesses and different energy characteristics of the surface, which were calculated in the framework of the Gibbs—Thompson theory. Based on the analysis of the surface energy values, it can be assumed that in the micro-shish-kebab structure, which made up about 45% of the total reactor powder, the surface of the lamellae was formed by regular folds, which introduced significant distortions into the structure of the crystalline core of the lamellae. About 7% of the macro-shish-kebabs had the same structure. The surface of the remaining lamellae in different morphoses was less ordered and was mostly formed by irregular folds and segments of molecular chains in various conformations. The assumption is made that the extended molecular segments and micro-shish kebabs incorporated in the central shish first formed in the local overheated regions. The formations of the morphoses were so different in their structural and energy characteristics during synthesis, apparently due to the heterogeneity of the thermal and shear fields in the reactor in which the polymer was synthesized. The polymer factually underwent a flow-induced non-isothermal crystallization process.

## Figures and Tables

**Table 1:** Parameters of the separated diffraction peaks.

Peak Numbers	1	2	3	4	5	6	7
2θ, degrees	0.133	0.198	0.327	0.418	0.540	0.679	0.824
L_SAXS,_ nm	66.3	44.6	27.0	21.0	16.3	13.0	10.7
Contribution to SAXS, %	7.2	11.0	44.7	32.9	2.9	0.6	0.6

**Table 2:** Values of the identified long periods, lamella thicknesses, and values of the surface energy for each component of the expansion of the SAXS curve.

L_SAXS_, nm	L_lam_, nm	l_interphase_ *, nm	Contribution to the Total SAXS Curve, %	σ_e_, 10^−3^ J/m^2^
10.7	6.5	0.15	0.6	33.6
13.0	7.9	0.85	0.6	40.8
16.3	9.9	1.85	2.9	51.2
21.1	12.9	3.35	32.9	66.7
27.0	16.5	5.15	44.7	85.3
44.6	10.3	2.05	11.0	53.3
66.3	15.2	4.50	7.2	78.6

* l_interphase_ = (L_lam_ − l_cr_)/2; l_cr_ = 6.2 nm.

## Data Availability

Not applicable.
